# Association of Behavioral Risk Factors for Chronic Diseases With Physical and Mental Health in European Adults Aged 50 Years or Older, 2004–2005

**DOI:** 10.5888/pcd12.150134

**Published:** 2015-09-17

**Authors:** Manolis Linardakis, Angeliki Papadaki, Emmanouil Smpokos, Katerina Micheli, Maria Vozikaki, Anastas Philalithis

**Affiliations:** Author Affiliations: Angeliki Papadaki, Centre for Exercise, Nutrition, and Health Sciences, School for Policy Studies, University of Bristol, United Kingdom, and Department of Social Medicine, Faculty of Medicine, University of Crete, Greece; Emmanouil Smpokos, Katerina Micheli, Maria Vozikaki, Anastas Philalithis, Department of Social Medicine, Faculty of Medicine, University of Crete, Greece.

## Abstract

**Introduction:**

Noncommunicable diseases are the leading cause of illness and death worldwide; behavioral risk factors (BRFs) contribute to these diseases. We assessed the presence of multiple BRFs among European adults according to their physical and mental health status.

**Methods:**

We used data from 26,026 adults aged 50 years or older from 11 countries that participated in the Survey of Health, Ageing and Retirement in Europe (2004–2005). BRFs (overweight or obesity, smoking, physical inactivity, and risky alcohol consumption) were assessed according to physical health (ie, presence of chronic diseases, disease symptoms, or limitations in activities of daily living) and mental health (depression) through multiple regression estimations.

**Results:**

Overweight or obesity in men and physical inactivity in women were the most prevalent BRFs. Compared with physically active adults, physically inactive adults had a higher mean number of chronic diseases (1.33 vs 1.26) and chronic disease symptoms (1.55 vs 1.47). Risky alcohol consumption (≥4 servings of an alcohol beverage ≥3 times a week) was associated with a higher mean depression score (2.84 vs 2.47). Compared with adults with 0 or 1 BRF, adults with 2 or more BRFs had significantly higher odds of having 1 or more chronic diseases (men: 1.52; women: 1.73) and functional limitations (men: 1.65; women: 1.79) and higher prevalence of high blood pressure (37.8% vs 28.2). Belgian adults with BRFs had the highest mean number of chronic diseases or functional limitations among those who were overweight or obese and the highest mean number of chronic diseases and disease symptoms among those who smoked and were physically inactive.

**Conclusion:**

We found revealed significant positive associations between BRFs and poor health among middle-aged and older European adults. Primary health care intervention programs should focus on developing ways to reduce BRF prevalence in this population.

## Introduction

Noncommunicable diseases (NCDs) are the leading causes of disease and death worldwide, and their symptoms and resulting functional limitations are related to impaired quality of life (1–5). NCDs accounted for 57 million deaths in 2008, of which 63% were attributed to cardiovascular disease, diabetes, cancer, and chronic respiratory disease (5).

Numerous lifestyle habits, identified as behavioral risk factors (BRFs), may increase NCD risk. These risk factors include overweight or obesity, smoking, physical inactivity, and risky alcohol consumption (2,4–8). Each of these risk factors alone can cause numerous health problems. For example, from 2008 to 2030 deaths attributed to smoking worldwide are expected to double, from 3.4 to 6.8 million (4,5,9). Overweight and obesity, direct consequences of physical inactivity and unhealthy diet, are responsible for 2.8 million deaths annually (4).

The greatest burden of disease and death related to BRFs from 2009 through 2011 occurred in countries of the World Health Organization European Region, the Eastern Mediterranean Region, and the Region of the Americas (4,5,9). The prevalence of BRFs varies among populations; clustering of 2 or more BRFs, an indication of increased risk for chronic diseases (10,11), occurs in approximately 57% of the adult population in the United States and 50% of Canadian adults aged 50 years or older (6,7,12).

Few large-scale studies examined the presence of BRFs in European adults according to physical and mental health. The aim of this study was to assess the presence of multiple BRFs in adults aged 50 years or older in 11 European countries, according to their physical and mental health status.

## Methods

Cross-sectional data were collected from 26,026 adults aged 50 years or older (range, 50–104 y), during the first wave (2004–2005) of SHARE (Survey of Health, Ageing, and Retirement in Europe) in 11 European countries (Austria, Belgium, Denmark, France, Germany, Greece, Italy, Netherlands, Spain, Sweden, and Switzerland). A subsample was selected in each country according to complex multistage stratification design. The target population consisted of households with at least 1 person aged 50 years or older. The overall weighted country-average response rate (households and individuals) was 61.8% and ranged from 37.6% (Switzerland) to 73.6% (France). Comparable differences in response rates have been reported in similar surveys (13). A detailed description of the sampling procedures, recruitment rates, and other survey features has been reported elsewhere (10,13–15).

### Data collection: questionnaires

Computer-aided personal interviews (CAPIs), consisting of 21 modules, were used to collect data in person (eg, demographic characteristics, physical and mental health, BRFs) (13). Proxy interviews (6.0% of all completed interviews) were allowed when physical or mental health limitations (eg, Alzheimer’s disease, hearing loss) prevented a selected participant from completing the CAPIs (13). Example show cards (ie, a page of the questionnaire with a list of diseases, symptoms, limitations, etc) were used in some modules to help participants understand the questions, enhancing the validity of the questionnaire (16). Data were missing from only 5% of the CAPIs.

### Physical and mental health assessment

Physical health was assessed by the presence of chronic diseases, disease symptoms, functional limitations, or disabilities during the previous 6 months; these features were recorded by using validated scales via personal interviews (13,17). To assess chronic diseases, participants were asked if a doctor had diagnosed any of 11 diseases: heart attack, high blood pressure, high blood cholesterol, stroke, diabetes or high blood glucose, chronic lung disease, asthma, arthritis, osteoporosis, cancer, and stomach or duodenal/peptic ulcer. To assess symptoms, participants were asked if they had any of 11 conditions: pain in back, knees, hips or other joints; heart trouble; breathlessness; persistent cough; swollen legs; sleeping problems; falls; fear of falling; dizziness, faints, or blackouts; stomach or intestine problems; and incontinence. Functional limitations of daily living, defined as activities and instrumental activities ([I]ADLs), were assessed by asking participants if they had limitations in any of 13 activities: dressing (including shoes and socks), walking across a room, bathing or showering, eating or cutting up food, getting in or out of bed, using the toilet (including getting up or down), using a map in a strange place, preparing a hot meal, shopping for groceries, making telephone calls, taking medications, doing work around the house or garden, or managing money. Mental health was assessed by using the European Depression (Euro-D) Scale, which defines clinically depressive symptoms by a total score of 4 or more in the 12-item validated questionnaire (17–19).

To assess physical and mental health status, the presence of 1 or more chronic diseases, disease symptoms, or (I)ADL limitations was defined separately for every component as its presence (= 1) or absence (= 0) or as a high score (≥4) in the depression scale. A total clustering score for physical and mental health was then calculated by summing the resulting binary variables for each adult. This clustering score ranged from 0 to 4, and 4 cluster categories were created, combining the 4 components as 0, 1 or 2, 3 or 4, or 1 or more of these components. This clustering score depicts the presence of multiple components as a higher burden on health or poor physical or mental health.

### Behavioral risk factors

Four health-related BRFs were assessed, namely overweight or obesity, smoking, physical inactivity, and risky alcohol consumption (6,7,10–12). Overweight or obesity were determined by self-reported body weight in kilograms and height in meters (14). Body mass index (BMI) was calculated as kg/m^2^, and participants with a BMI of 25 or greater were considered overweight or obese (4). Smoking was assessed from self-reported use of cigarettes, cigars, or pipes during the year preceding the survey. To determine physical activity levels, activities, activities such as gardening or walking were considered moderate physical activities, whereas activities such as sports or heavy home labor were considered vigorous physical activity. Frequency of physical activity was classified as more than once per week, once per week, 1 to 3 times per month, or hardly ever or never. Physical inactivity was defined as not engaging in any moderate-to-vigorous physical activity or having a low frequency of physical activity (once a week, 1 to 3 times a month, or hardly ever or never) (7,10–13). Risky alcohol consumption was defined as the consumption of 4 or more servings of alcoholic beverages on at least 3 days a week during the 6 months preceding the survey (7,10,11,13).

The clustering of BRFs was estimated by adding the number of individual factors that were present (0 = absence, 1 = presence) to create an average clustering or mean factors score, ranging from 0 to 4. The clustering of 2 or more risk factors was considered to depict high risk for chronic disease (10,11).

### Self-rated health

Participants self-rated their health by using the World Health Organization scale, reporting health as very good, good, fair, or bad or very bad (17).

### Socioeconomic characteristics

The social and demographic variables of age, living status, retirement, and educational status were assessed. Living status consisted of 2 categories, living alone and living with a partner or spouse. Retirement consisted of 2 categories (yes or no) and educational status was calculated as total years of schooling (10). Financial status was assessed as the gross household income in the previous year (13,20).

### Statistical analysis

Data were analyzed using SPSS software, version 21.0 (IBM Corp). Weights were applied to reflect nonresponses and stratification design. The prevalence of individual and clustering (0, 1 or 2, 3 or 4, or ≥1) of physical and mental health components and BRFs was estimated with the corresponding 95% confidence intervals (CIs). Weighted means of BRFs and their 95% CIs were estimated for each cluster category of physical and mental health status for each sex by using analysis of covariance according to complex sample design procedures. Age, education, living status, country region (north, central, south) (11), self-rated health, income, and retirement status were used as covariates. Multiple logistic regression analysis (using the same covariates) was conducted to compute adjusted odds ratios (ORs) of participants who had 1 or more chronic diseases, disease symptoms, or (I)ADL limitations; Euro-D scores of 4 or more; or clustering (1 or 2, 3 or4, or ≥1) of physical or mental health components. Adjusted ORs were estimated separately for the presence of 1 BRF and 2 or more BRFs. Differences in mean numbers of chronic diseases, symptoms, (I)ADL limitations, and Euro-D Scale scores were assessed, and analysis of covariance was used to estimate weighted means according to presence or absence of individual BRFs. Age, sex, education, living status, country region, self-rated health, income, and retirement status were used as covariates. Finally, prevalence of all chronic diseases, disease symptoms, and (I)ADL limitations according to clustering of BRFs were illustrated as weighted percentages and 95% CIs.

## Results

Mean age and years of education of participants were 65.2 and 9.7 years, respectively (Table 1). A greater percentage of men than women were living with a partner or spouse or were retired, and a smaller percentage of men self-rated their health as bad or very bad. Overweight or obesity, smoking, and risky alcohol consumption were more prevalent in men. Physical inactivity was more prevalent in women. The prevalence of 2 or more BRFs was significantly higher among men (58.4%; 95% CI, 57.1%–60.0%) than among women (49.0%; 95% CI, 47.7%–50.4%). In contrast, men had significantly lower prevalence of 1 or more chronic diseases, disease symptoms, and (I)ADL limitations, and a score or 4 or more on the Euro-D Scale. Men also had higher prevalence of having 0 components of physical and mental health (17.1%; 95% CI,16.0%–18.2%), compared with women (11.2%; 95% CI,10.4%–12.0%).

**Table 1:** Characteristics, Physical and Mental Health Status Components, and Behavioral Risk Factors for Chronic Disease Among European Adults (n = 26,026) Aged 50 Years or Older, Survey of Health, Ageing, and Retirement in Europe, 2004–2005^a^

Characteristic	Total (n = 26,026)	Men (n = 12,030)	Women (n = 13,996)
Age, y, weighted mean (SD)	65.2 (10.4)	64.1 (9.8)	66.2 (10.8)
Education, y, weighted mean (SD)	9.7 (4.9)	10.4 (4.9)	9.1 (4.8)
**Living status**			
Lives alone	33.2	21.0 (19.7–22.5)	43.6 (42.2–45.0)
Lives with partner or spouse	66.8	79.0 (77.5–80.3)	56.4 (55.0–57.8)
**Retirement status**			
Retired	50.9	58.1 (56.6–59.6)	44.7 (43.4–46.1)
**Self-rated health**			
Bad or very bad	11.7	10.5 (9.6–11.5)	12.6 (11.7–13.7)
**Components of physical and mental health status^c^**			
Chronic diseases^b^, ≥1	67.7	64.6 (63.2–66.0)	70.2 (69.0–71.5)
Disease symptoms^c^, ≥1	68.9	62.0 (60.6–63.4)	73.6 (71.8 –75.9)
(I)ADL^d^ limitations, ≥1	20.5	16.7 (15.6–17.8)	23.8 (22.6–25.1)
**European Depression Scale^e^**			
Score ≥4	26.6	19.3 (18.1–20.5)	32.7 (31.4–34.1)
**No. of health status components**			
0	13.9	17.1 (16.0–18.2)	11.2 (10.4–12.0)
1 or 2	58.8	63.1 (61.6–64.5)	55.2 (53.8–56.6)
3 or 4	27.3	19.9 (18.7–21.1)	33.6 (32.3–34.9)
**Behavioral risk factor**			
Overweight or obese^f^	60.0	66.7 (65.3–68.1)	54.2 (52.8–55.6)
Smoker^g^	18.3	24.3 (23.0–25.6)	13.1 (12.3–14.1)
Physical inactivity^h^	70.8	65.9 (64.5–67.3)	75.0 (73.9–76.2)
Risky alcohol consumption^i^	4.3	8.0 (7.2–8.8)	1.3 (1.0–1.6)
**No. of behavioral risk factors**			
0	9.2	8.1 (7.4–8.9)	10.2 (9.4–11.0)
1	37.4	33.4 (32.0–34.8)	40.7 (39.4–42.1)
≥2	53.4	58.4 (57.1–60.0)	49.0 (47.7–50.4)

Abbreviation: (I)ADL, activities and instrumental activities of daily living.

a Values are weighted percentage and confidence intervals unless otherwise noted.

b Chronic diseases refer to the following chronic diseases: heart attack, high blood pressure, high blood cholesterol, stroke, diabetes or high blood glucose, chronic lung disease, asthma, arthritis, osteoporosis, cancer, and stomach or duodenal/peptic ulcer.

c Symptoms refer to the following: pain in back, knees, hips or other joints; heart trouble; breathlessness; persistent cough; swollen legs; sleeping problems; falls; fear of falling down; dizziness; faints or blackouts; stomach or intestine problems; and incontinence.

d (I)ADL refers to having a limitation in the following activities: dressing (including shoes and socks), walking across a room, bathing or showering, eating, cutting up food, getting in or out of bed, using the toilet (including getting up or down), using a map in a strange place, preparing a hot meal, shopping for groceries, making telephone calls, taking medications, doing work around the house or garden, and managing money.

e The European Depression Scale was used to define clinically depressive symptoms, as indicated by a total score of ≥4 symptoms in the 12-item validated questionnaire.

f Overweight or obesity were determined by self-reported body weight in kilograms and height in meters. Body mass index (BMI) was calculated as kg/m^2^, and participants with a BMI of ≥25 were considered overweight or obese.

g Smoking was assessed from self-reported use of cigarettes, cigars, or pipes during the year preceding the survey.

h Physical inactivity was defined as the lack of weekly engagement in moderate-to-vigorous activities (per week) during the research period. Activities such as gardening or walking were considered moderate physical activities, whereas activities such as sports or heavy home labor were considered vigorous physical activity. Frequency was classified as less than once per week, once per week, 1 to 3 times per month, or hardly ever or never. Physical inactivity was defined as not engaging in any moderate-to-vigorous physical activity or having low frequency of physical activity (once per week, 1 to 3 times per month, or hardly ever or never).

i Risky alcohol consumption was defined as the consumption of 4 or more servings of alcoholic beverages on at least 3 days a week during the 6 months preceding the survey.

Both men and women with 1 or more chronic diseases or 1 or more (I)ADL limitations had significantly greater weighted mean numbers of BRFs than those with none (Table 2). Respectively, the adjusted ORs in men with 2 or more BRFs were higher for those with 1 or more chronic diseases (1.52; 95% CI, 1.20–1.91) or (I)ADL limitations (1.65; 95% CI,1.10–2.48). Women with 2 or more BRFs also had higher odds of having 1 or more chronic diseases (1.73; 95% CI,1.42–2.12), disease symptoms (1.39; 95% CI,1.10–1.72), or (I)ADL limitations (1.79; 95% CI,1.30–2.43); they also had higher odds of having any of the 3 physical and mental health status cluster component combinations than women with 1 BRF (1 or 2 components: 1.34 [95% CI,1.04–1.74]; 3 or 4 components: 1.86 [95% CI,1.28–2.69]; ≥1 components: 1.43 [95% CI,1.11–1.84]).

**Table 2:** Clustering of Behavioral Risk Factors According to Presence of Physical and Mental Health Components in European Adults (n = 26,026) Aged 50 Years or Older, Survey of Health, Ageing, and Retirement in Europe, 2004–2005

Characteristic	Number	Behavioral Risk Factors		
		Weighted Mean (95% CI)	Adjusted Odds Ratio^a^ (95% CI)^b^	
			1	≥2
**Men**				
Chronic diseases	0	1.59 (1.55–1.64)	Reference	
	≥1	1.68 (1.65–1.71)	1.28 (1.01–1.62)	1.52 (1.20–1.91)
Disease symptoms	0	1.61 (1.57–1.66)	Reference	
	≥1	1.67 (1.64–1.70)	0.99 (0.78–1.25)	1.17 (0.93–1.47)
(I)ADL limitations	0	1.63 (1.61–1.66)	Reference	
	≥1	1.73 (1.67–1.79)	1.36 (0.88–2.12)	1.65 (1.10–2.48)
Euro-D Scale score	<4	1.64 (1.62–1.67)	Reference	
	≥4	1.67 (1.61–1.72)	1.21 (0.86–1.72)	1.26 (0.91–1.75)
Health status components	0	1.59 (1.52–1.65)	Reference	
	1–2	1.64 (1.61–1.67)	1.05 (0.80–1.38)	1.27 (0.98–1.66)
	3–4	1.74 (1.67–1.80)	1.13 (0.66–1.92)	1.68 (1.01–2.78)
	≥1	1.66 (1.64–1.69)	1.04 (0.80–1.36)	1.29 (0.99–1.68)
**Women**				
Chronic diseases	0	1.33 (1.29–1.37)	Reference	
	≥1	1.48 (1.46–1.51)	1.30 (1.07–1.58)	1.73 (1.42–2.12)
Disease symptoms	0	1.36 (1.31–1.40)	Reference	
	≥1	1.47 (1.44–1.49)	1.20 (0.98–1.47)	1.39 (1.10–1.72)
(I)ADL limitations	0	1.42 (1.39–1.44)	Reference	
	≥1	1.51 (1.47–1.55)	1.43 (1.04–1.98)	1.79 (1.30–2.43)
European Depression Scale score	<4	1.44 (1.41–1.46)	Reference	
	≥4	1.44 (1.40–1.48)	1.01 (0.80–1.27)	1.09 (0.87–1.36)
Health status components	0	1.32 (1.26–1.38)	Reference	
	1 or 2	1.39 (1.36–1.41)	1.18 (0.92–1.50)	1.34 (1.04–1.74)
	3 or 4	1.55 (1.51–1.58)	1.36 (0.95–1.95)	1.86 (1.28–2.69)
	≥1	1.45 (1.43–1.47)	1.21 (0.95–1.54)	1.43 (1.11–1.84)

Abbreviations: CI, confidence interval; (I)ADL, activities and instrumental activities of daily living.

a In relation to having 0 behavioral risk factors.

b Confidence intervals are based on analysis of covariance and logistic regression analysis (using complex sample design procedure). In both methods, age, education, living with a partner or spouse, country region (north, central, south), self-rated health, income, and retirement status were used as covariates.

Overweight or obese participants had a higher mean number of chronic diseases (1.43 vs 1.13, *P* < .001) and disease symptoms (1.62 vs. 1.39, *P* < .001) than participants of normal weight, whereas smokers had a lower mean number of chronic diseases (1.23 vs. 1.32, *P* = .005) than nonsmokers (Table 3). Physically inactive participants had a higher mean number of chronic diseases (1.33 vs. 1.26, *P* = .009), disease symptoms (1.55 vs 1.47, *P* = .01), and (I)ADL limitations (0.63 vs 0.44, *P* < .001) than physically active participants. Risky drinkers had a higher mean score on the Euro-D Scale (2.84 vs 2.47, *P* = .003), compared with nonrisky drinkers.

**Table 3:** Chronic Diseases, Disease Symptoms, (I)ADL Limitations, and Depression Score According to Presence or Absence of Behavioral Risk Factors Among European Adults (n = 26,026) Aged 50 Years or Older, Survey of Health, Ageing, and Retirement in Europe, 2004–2005

Behavioral Risk Factor	Physical and Mental Health Status Components, Weighted Mean (Standard Error)^a^			
	Chronic Diseases	Disease Symptoms	(I)ADL Limitations	Euro-D Score
**Body weight^b^**				
Overweight or obese	1.43 (0.01)	1.62 (0.02)	0.55 (0.02)	2.45 (0.03)
Normal^c^	1.13 (0.02)	1.39 (0.02)	0.62 (0.03)	2.55 (0.03)
*P* value	<.001	<.001	.07	.03
**Smoking status^d^**				
Smoker	1.23 (0.02)	1.55 (0.03)	0.57 (0.03)	2.51 (0.05)
Nonsmoker or former smoker	1.32 (0.01)	1.53 (0.01)	0.57 (0.02)	2.49 (0.02)
*P* value	.005	.53	.87	.61
**Physically inactive^e^**				
Yes	1.33 (0.01)	1.55 (0.02)	0.63 (0.02)	2.51 (0.02)
No	1.26 (0.02)	1.47 (0.02)	0.44 (0.02)	2.43 (0.04)
*P* value	.009	.01	<.001	.09
**Alcohol consumption^f^**				
Risky drinker	1.28 (0.05)	1.50 (0.05)	0.50 (0.05)	2.84 (0.09)
Nonrisky drinker	1.31 (0.01)	1.53 (0.01)	0.58 (0.02)	2.47 (0.02)
*P* value	.58	.62	.17	.003

Abbreviations: Euro-D, European Depression Scale; (I)ADL, activities and instrumental activities of daily living.

a Comparisons were made by using analysis of covariance (according to the complex sample design procedure), with sex, age (y), education (y), living with a partner or spouse, country regions (north, central, south), self-rated health, income, and retirement status as covariates.

b Overweight or obesity were determined by self-reported body weight in kilograms and height in meters. Body mass index (BMI) was calculated as kg/m^2^, and participants with a BMI of ≥25 were considered overweight or obese.

c Normal weight was BMI<25.

d Smoking was assessed from self-reported use of cigarettes, cigars, or pipes during the year preceding the survey.

e Physical inactivity was defined as the lack of weekly engagement in moderate-to-vigorous activities (per week) during the research period. Activities such as gardening or walking were considered moderate physical activities, whereas activities such as sports or heavy home labor were considered vigorous physical activity. Frequency was classified as less than once per week, once per week, 1 to 3 times per month, or hardly ever or never. Physical inactivity was defined as not engaging in any moderate-to-vigorous physical activity or having a low frequency of physical activity (once per week, 1 to 3 times per month, or hardly ever or never).

f A risky drinker was defined a person who consumed 4 or more servings of alcoholic beverages on at least 3 days per week during the 6 months preceding the survey. A nonrisky drinker was defined as a person who consumed fewer than 4 servings of alcoholic beverages 3 days a week.

**Figure 1:**
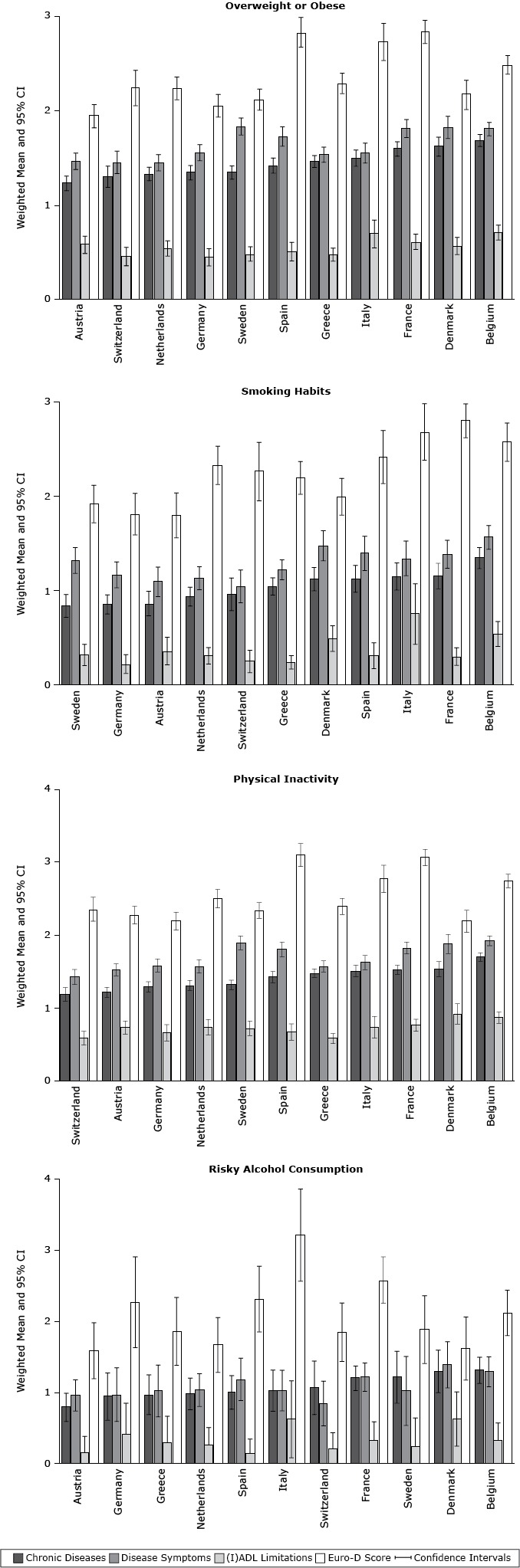
Weighted mean number of physical and mental health status components among participants with different behavioral risk factors in 11 European countries, Survey of Health, Ageing and Retirement in Europe, 2004–2005. Comparisons were examined using analysis of covariance (according to the complex sample design procedure), with sex, age (y), education (y), living with a partner or spouse, self-rated health, income, and retirement status as covariates. Abbreviations: CI, confidence interval; (I)ADL, activities and instrumental activities of daily living; Euro-D score, European Depression Scale Score. **Table:** 

Belgian adults had the highest mean number of chronic diseases and (I)ADL limitations among overweight or obese participants (Figure 1) and the highest mean number of chronic diseases and disease symptoms among participants who smoked and were physically inactive and had the highest number of chronic diseases among risky alcohol drinkers. In contrast, among participants who were overweight or obese, Austrian adults had the lowest mean number of chronic diseases (1.24; 95% CI, 1.16–1.31). Austrian adults also had the lowest risky alcohol consumption, and among those with risky alcohol consumption, the lowest mean Euro-D score (1.59; 95% CI, 1.19–1.98).

Country	Chronic Disease, Mean (95% CI)	Disease Symptoms, Mean (95% CI)	(I)ADL Limitations, Mean (95% CI)	Euro-D Score, Mean (95% CI)
**Overweight or obese**				
Austria	1.24 (1.16–1.31)	1.47 (1.38–1.56)	0.58 (0.49–0.68)	1.95 (1.83–2.07)
Switzerland	1.31 (1.19–1.42)	1.46 (1.34–1.58)	0.46 (0.36–0.56)	2.25 (2.06–2.44)
Netherlands	1.34 (1.27–1.41)	1.46 (1.37–1.54)	0.54 (0.46–0.63)	2.24 (2.12–2.37)
Germany	1.35 (1.27–1.43)	1.56 (1.48–1.65)	0.45 (0.36–0.54)	2.06 (1.94–2.18)
Sweden	1.35 (1.28–1.42)	1.84 (1.75 –1.93)	0.49 (0.41 –0.56)	2.13 (2.01 –2.24)
Spain	1.43 (1.35–1.51)	1.73 (1.63 –1.84)	0.51 (0.41 –0.61)	2.84 (2.68 –3.00)
Greece	1.47 (1.41–1.53)	1.54 (1.46–1.62)	0.48 (0.41–0.55)	2.30 (2.19–2.41)
Italy	1.50 (1.42–1.59)	1.56 (1.45–1.66)	0.70 (0.55–0.85)	2.74 (2.54–2.94)
France	1.60 (1.53–1.68)	1.82 (1.72–1.91)	0.62 (0.54–0.70)	2.85 (2.72–2.97)
Denmark	1.63 (1.52–1.73)	1.83 (1.71–1.95)	0.57 (0.48–0.66)	2.18 (2.02–2.33)
Belgium	1.69 (1.63–1.76)	1.81 (1.74–1.88)	0.71 (0.63–0.79)	2.50 (2.40–2.59)
**Smoking habits**				
Sweden	0.84 (0.72–0.96)	1.33 (1.19–1.46)	0.32 (0.21–0.43)	1.93 (1.72–2.13)
Germany	0.86 (0.76–0.96)	1.17 (1.03–1.31)	0.23 (0.13–0.33)	1.82 (1.60–2.04)
Austria	0.87 (0.74–1.00)	1.10 (0.94–1.26)	0.36 (0.22–0.51)	1.80 (1.57–2.04)
Netherlands	0.94 (0.84–1.04)	1.14 (1.01–1.26)	0.31 (0.23–0.40)	2.34 (2.13–2.54)
Switzerland	0.97 (0.79–1.14)	1.05 (0.88–1.23)	0.25 (0.13–0.37)	2.27 (1.96–2.58)
Greece	1.05 (0.96–1.14)	1.23 (1.12–1.33)	0.24 (0.17–0.31)	2.20 (2.03–2.38)
Denmark	1.13 (1.00–1.25)	1.48 (1.32–1.64)	0.50 (0.36–0.63)	2.00 (1.81–2.20)
Spain	1.13 (0.99–1.27)	1.40 (1.22–1.58)	0.31 (0.18–0.45)	2.42 (2.14–2.71)
Italy	1.15 (1.01–1.30)	1.35 (1.16–1.53)	0.76 (0.44–1.08)	2.69 (2.35–3.03)
France	1.16 (1.02–1.30)	1.39 (1.24–1.54)	0.30 (0.21–0.40)	2.81 (2.55–3.06)
Belgium	1.35 (1.24–1.46)	1.57 (1.45–1.70)	0.54 (0.41–0.68)	2.58 (2.38–2.79)
**Physical inactivity**				
Switzerland	1.20 (1.11–1.29)	1.44 (1.33–1.54)	0.60 (0.50–0.69)	2.37 (2.20–2.54)
Austria	1.23 (1.16–1.30)	1.54 (1.46–1.62)	0.74 (0.65–0.83)	2.29 (2.17–2.41)
Germany	1.30 (1.23–1.37)	1.60 (1.51–1.69)	0.67 (0.56–0.78)	2.21 (2.08–2.33)
Netherlands	1.32 (1.26–1.39)	1.58 (1.49–1.68)	0.75 (0.64–0.85)	2.52 (2.39–2.64)
Sweden	1.33 (1.26–1.40)	1.91 (1.81–2.00)	0.73 (0.63–0.83)	2.35 (2.24–2.46)
Spain	1.44 (1.36–1.52)	1.82 (1.71–1.92)	0.68 (0.57–0.79)	3.11 (2.96–3.27)
Greece	1.49 (1.43–1.55)	1.58 (1.50–1.66)	0.59 (0.52–0.66)	2.41 (2.30–2.52)
Italy	1.52 (1.44–1.60)	1.64 (1.54–1.74)	0.75 (0.60–0.90)	2.79 (2.60–2.98)
France	1.54 (1.47–1.60)	1.84 (1.76–1.92)	0.77 (0.69–0.86)	3.08 (2.97–3.20)
Denmark	1.54 (1.44–1.65)	1.89 (1.76–2.02)	0.93 (0.79–1.07)	2.21 (2.05–2.36)
Belgium	1.71 (1.65–1.77)	1.94 (1.87–2.00)	0.88 (0.80–0.96)	2.76 (2.67–2.85)
**Risky alcohol consumption**				
Austria	0.79 (0.59–0.99)	0.96 (0.74–1.18)	0.16 (0.04–0.29)	1.59 (1.19–1.98)
Germany	0.94 (0.61–1.27)	0.97 (0.59–1.35)	0.42 (0.08–0.76)	2.27 (1.63–2.91)
Greece	0.97 (0.69–1.25)	1.02 (0.66–1.38)	0.29 (0.01–0.57)	1.86 (1.38–2.34)
Netherlands	0.98 (0.76–1.20)	1.03 (0.80–1.26)	0.26 (0.11–0.41)	1.67 (1.28–2.06)
Spain	1.00 (0.76–1.24)	1.18 (0.88–1.48)	0.13 (0.02–0.25)	2.32 (1.85–2.78)
Italy	1.02 (0.74–1.31)	1.03 (0.74–1.31)	0.62 (0.08–1.16)	3.22 (2.43–4.01)
Switzerland	1.06 (0.69–1.44)	0.84 (0.53–1.16)	0.20 (0.06–0.34)	1.85 (1.44–2.26)
France	1.20 (1.03–1.37)	1.22 (1.02–1.42)	0.33 (0.16–0.49)	2.58 (2.26–2.91)
Sweden	1.21 (0.85–1.58)	1.02 (0.53–1.51)	0.23 (0.08–0.55)	1.88 (1.41–2.36)
Denmark	1.30 (1.00–1.60)	1.39 (1.06–1.71)	0.62 (0.24–1.01)	1.62 (1.17–2.06)
Belgium	1.31 (1.13–1.50)	1.29 (1.08–1.50)	0.32 (0.16–0.48)	2.12 (1.80–2.44)

**Figure 2:**
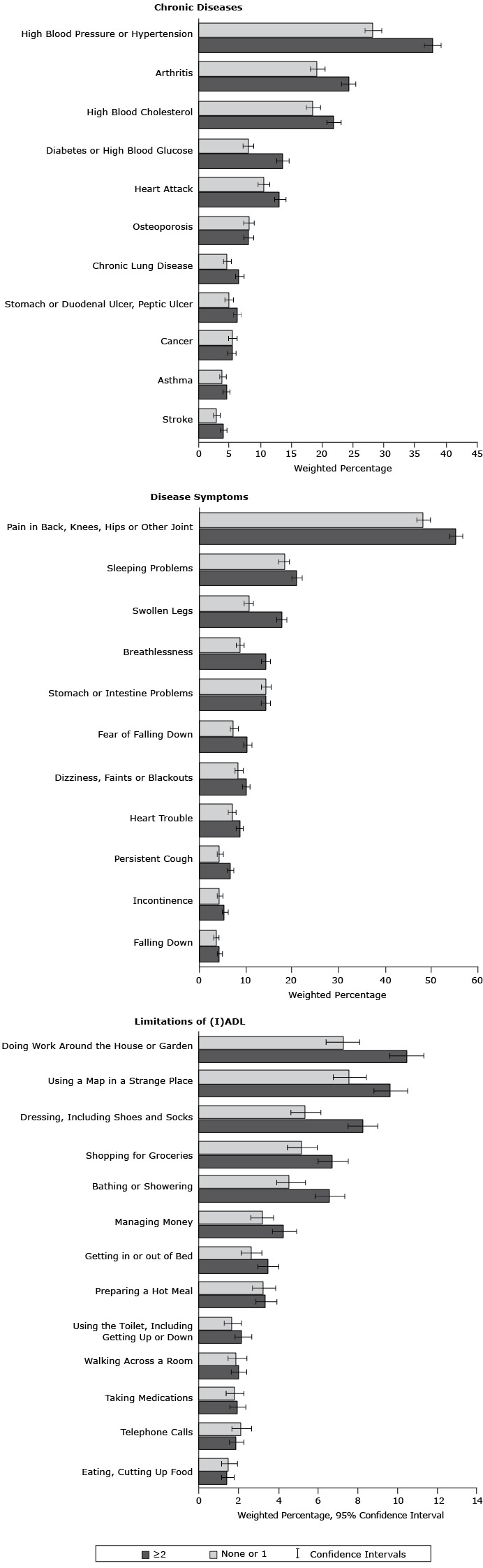
Prevalence of chronic diseases, disease symptoms, and limitations of activities and instrumental activities of daily living according to clustering score of behavioral risk factors. , Survey of Health, Ageing and Retirement in Europe, 2004–2005. Abbreviation: (I)ADL, limitations of activities and instrumental activities of daily living. **Table:** 

Participants with 2 or more BRFs, compared with those with none or 1 BRF, had significantly higher prevalence rates of high blood pressure (37.8%; 95% CI, 36.4%–39.1% vs 28.2%; 95% CI, 26.9%–29.6%) (Figure 2) and pain in back, knees, hips or other joints (55.3%; 95% CI, 54.0%–56.7% vs 48.3%; 95% CI, 46.8%–49.8%).

Characteristic	No. of Behavioral Risk Factors	
	≥2, % (95% Confidence Interval)	≤1, % (95% Confidence Interval)
**Chronic diseases**		
Stroke	4.0 (3.5–4.6)	2.9 (2.4–3.5)
Asthma	4.5 (3.9–5.1)	3.9 (3.4–4.5)
Cancer	5.4 (4.7–6.0)	5.5 (4.9–6.2)
Stomach or duodenal ulcer, peptic ulcer	6.2 (5.6–6.9)	4.9 (4.3–5.6)
Chronic lung disease	6.6 (6.0–7.4)	4.6 (4.0–5.3)
Osteoporosis	8.1 (7.3–8.9)	8.1 (7.3–9.0)
Heart attack	13.2 (12.3–14.1)	10.5 (9.6–11.5)
Diabetes or high blood glucose	13.6 (12.6–14.6)	8.0 (7.2–8.9)
High blood cholesterol	21.8 (20.7–23.0)	18.5 (17.4–19.7)
Arthritis	24.2 (23.0–25.4)	19.2 (18.0–20.4)
High blood pressure	37.8 (36.4–39.1)	28.2 (26.9–29.6)
**Disease symptoms**		
Falling	4.4 (3.9–5.0)	3.6 (3.1–4.2)
Incontinence	5.5 (5.0–6.2)	4.4 (3.9–5.1)
Persistent cough	6.7 (6.0–7.4)	4.5 (3.9–5.2)
Heart trouble	8.6 (7.9–9.5)	7.0 (6.2–7.9)
Dizziness, faints or blackouts	10.1 (9.3–10.9)	8.5 (7.7–9.5)
Fear of falling	10.4 (9.6–11.3)	7.5 (9.7–8.4)
Stomach or intestine problems	14.3 (13.3–15.3)	14.4 (13.4–15.5)
Breathlessness	14.3 (13.4–15.3)	8.7 (7.9–9.6)
Swollen legs	17.7 (16.7–18.8)	10.6 (9.6–11.6)
Sleeping problems	21.0 (19.9–22.2)	18.2 (17.1–19.4)
Pain in back, knees, hips, or other joint	55.3 (54.0–56.7)	48.3 (46.8–49.8)
**Limitations of activities and instrumental activities of daily living**		
Eating, cutting up food	1.4 (1.1–1.7)	1.4 (1.1–1.9)
Making telephone calls	1.8 (1.5–2.2)	2.1 (1.6–2.6)
Taking medications	1.9 (1.5–2.3)	1.7 (1.3–2.2)
Walking across a room	1.9 (1.6–2.4)	1.8 (1.4–2.4)
Using the toilet, including getting up or down	2.2 (1.8–2.6)	1.6 (1.2–2.1)
Preparing a hot meal	3.3 (2.8–3.9)	3.2 (2.6–3.8)
Getting in or out of bed	3.4 (2.9–4.0)	2.6 (2.1–3.1)
Managing money	4.2 (3.7–4.9)	3.1 (2.6–3.7)
Bathing or showering	6.5 (5.8–7.3)	4.5 (3.9–5.3)
Shopping for groceries	6.7 (6.0–7.5)	5.1 (4.4–5.9)
Dressing, including shoes and socks	8.2 (7.5–9.0)	5.3 (4.6–6.1)
Using a map in a strange place	9.6 (8.8–10.5)	7.5 (6.7–8.4)
Doing work around the house or garden	10.4 (9.6–11.3)	7.2 (6.4–8.1)

## Discussion

We examined the presence of BRFs in European adults aged 50 years or older, according to their physical and mental health status. The main findings were 1) the most prevalent BRFs were overweight or obesity in men and physical inactivity in women; 2) prevalence of 2 or more BRFs was higher in men, and prevalence of physical and mental health status components was lower in men; 3) men with 2 or more BRFs had higher odds for having 1 or more chronic diseases and (I)ADL limitations, and women with 2 or more BRFs had higher odds for having 1 or more of all health status components; 4) physically inactive adults had higher mean numbers of chronic diseases, disease symptoms, and (I)ADL limitations; 5) adults from Belgium with BRFs had the poorest physical health status among the 11 countries studied; and 6) among adults with 2 or more BRFs, high blood pressure was the most prevalent disease.

Our findings agree with earlier literature suggesting that men generally have a higher prevalence of BRFs for chronic disease (7,8,10,12,21). The findings of our study also indicate a positive relationship between the presence of BRFs and physical and mental health status components; women had 2 or more BRFs, displaying higher presence and clustering of these components. Women in our study had a higher prevalence of chronic disease, disease symptoms, and functional disabilities than men; however, men had a higher prevalence of BRFs. We cannot explain the cause–effect relationship of BRFs and health because of differences in health status among men and women. Women seem to have more health problems but men have more BRFs (16). The National Health Interview Survey, 2001, of adults aged 18 years or older in the United States also found that people with 2,3, or 4 risk factors had significantly higher odds of having a chronic disease (7). In a similar manner, a 2007 study of the Chinese population aged 15 to 69 years with chronic disease reported a significantly higher odds of BRF clustering as well as a significantly greater weighted mean number of BRFs (21). In addition, the survey of the Australian Institute of Health and Welfare (2007–2008) showed that an increasing number of BRFs was associated with increased odds of arthritis, ischemic heart disease, stroke, depression, and chronic obstructive pulmonary disease (22). Our study also showed that participants with 2 or more BRFs had significantly higher odds of having (I)ADL limitations than those with 0 or 1 BRF. The 2010 national Behavioral Risk Factor Surveillance System survey conducted in US adults aged 18 years or older (23) showed that participants with disabilities had higher odds of being both physically inactive, obese, or smokers, confirming the relationship between (I)ADL limitations and BRFs. These findings indicate the relationship between multiple BRFs and physical health and the necessity of prevention programs to focus on reducing BRF prevalence.

Our study showed that overweight or obesity and physical inactivity, which were present in more than half of adults participating in the SHARE survey, were the most prevalent BRFs. An earlier analysis of the same data also showed that women exhibited lower prevalence of overweight or obesity, smoking, and risky alcohol consumption, but higher prevalence of physical inactivity, than men and adults aged 80 years or older (10). Sufficient levels of physical activity have generally been shown to be protective against the development of chronic diseases (eg, cardiovascular disease) but may also protect mental health by reducing anxiety or depression (22). Thus, the high prevalence of physical inactivity in our sample could explain the high observed weighted mean numbers of chronic diseases, disease symptoms, (I)ADL limitations, and depression. In contrast, the potentially controversial finding of the association between normal weight status and higher depression score may be explained by the relationship between weight loss and depression (24). Additionally, overweight and obesity are known risk factors for high blood pressure, a biomedical risk factor with the highest contribution to chronic disease burden and mortality (22). Overweight or obesity was the second most prevalent BRF in the current study, which could explain our finding that high blood pressure was the most prevalent disease, particularly among participants with 2 or more BRFs. Our study showed that overweight or obese adults also had a higher mean number of symptoms of aging, such as pain in the back, knees, hips or other joints, confirming the reported association between overweight and obesity and these symptoms (25).

The association of BRFs with mortality has been widely documented. For example, in their recent systematic review, Loef and Walach (3) estimated that the adoption of 4 or more healthy lifestyle behaviors is associated with a 66% decrease in mortality risk. The Health and Retirement Study, which studied Americans aged 50 years or older, also showed that the combination of multiple unhealthy lifestyle factors was related to increased risk of mortality (26). This risk was 1.9 times higher for smokers, independently of other factors, and 4.0 times higher for participants with the combined behaviors of smoking and physical inactivity and heavy drinking. These findings are crucial for emphasizing the need for national agencies, organizations, and European governments to identify ways to address and prevent their relative consequences. In our study, however, smokers exhibited a lower number of chronic diseases than nonsmokers and ex-smokers. This might be explained by the fact that smokers in our study were younger (10), and older age is often associated with smoking cessation due to disease burden (22,27). Nevertheless, the increased mortality risk appears to be associated with the lifetime of smoking (27). Other effective interventions that could contribute to a decrease in the prevalence of some BRFs are the imposition of high taxation on tobacco products, alcohol, and foods with high energy density; clean indoor air laws; and advertising restrictions or mass media campaigns promoting health lifestyle behaviors (28,29). These efforts should be country-specific due to differences in income and social and health inequalities among countries (6).

Comparisons of countries revealed that adults from Belgium with BRFs had the poorest physical health status. An earlier report of the SHARE survey also showed that Belgians had a high prevalence of 2 or more BRFs (56.2%), although this prevalence was lower than that among their Austrian, Greek, and Spanish counterparts (11). Nevertheless, both this earlier report and the current study showed that less than 50% of adults had 2 or more BRFs, which was associated with high odds of poor physical and mental health, especially among women.

Our study has various methodological weaknesses that limit its external validity. The association of BRFs with physical and mental health was based on a cross-sectional design and therefore cannot be substantiated as a causal relationship. Similar studies, however, have also shown associations between individual BRFs and chronic diseases and disabilities (3,7,21–23), as well as prospective associations with mortality rates (3,26,27). In addition, it is difficult to compare findings among studies because of the different definitions of BRFs used in different studies. Self-reported body weight or height can also differ from objective measurements, and physical inactivity was estimated by using approximate cut-off points that might restrict assessment in older adults (10). Chronic diseases were assessed by asking participants if they had had a disease diagnosed. Ideally, future studies should include a validation of self-reported data by conducting objective measurements in a subsample of the population. Nevertheless, similar large-scale studies have also based their results on self-reported data (7,12,16,17). Also, risky alcohol consumption was not estimated by sex due to the use of fixed, closed-ended questions for both sexes. Assessment of smoking prevalence was based only on smoking occurrence and not on total burden of this behavior (eg, pack-years) (7,10,13,27). Likewise, the 11 chronic diseases included in the physical health status definition did not have the same burden on health. Furthermore, presence of dementia was not assessed during the first wave. Finally, our analysis did not allow the examination of the association of BRFs with individual chronic diseases (16,22). Nevertheless, we combined a large number of physical and mental health components, thereby adding to the literature examining the associations of BRFs with physical and mental health.

This study of middle-aged and older European adults showed that significant positive associations exist between unhealthy lifestyle behaviors and poor physical and mental health. Primary health promotion program should focus on examining whether any causal relationships exist and if so, identify ways to reduce BRFs, taking into account that interventions should consider the health care systems in individual countries (6,30). Such interventions could be applied at the community or home level or in primary health care and should involve patients and health care providers at all stages of their development and implementation to increase their chance of effectiveness. These program should focus on adults with multiple BRFs and offer them individualized care and guidance according to personal motivations, skills, and barriers for lifestyle changes, and should support use of screening and preventive health services to reduce BRFs (6,30).
